# Molecular Epidemiology of *Clostridium difficile* Infection in a Large Teaching Hospital in Thailand

**DOI:** 10.1371/journal.pone.0127026

**Published:** 2015-05-22

**Authors:** Popchai Ngamskulrungroj, Sittinee Sanmee, Papanin Pusathit, Pipat Piewngam, Briony Elliott, Thomas V. Riley, Pattarachai Kiratisin

**Affiliations:** 1 Department of Microbiology, Faculty of Medicine, Siriraj Hospital, Mahidol University, Bangkok, Thailand; 2 School of Pathology & Laboratory Medicine, The University of Western Australia, Crawley, Australia; 3 PathWest Laboratory Medicine WA, Queen Elizabeth II Medical Centre, Nedlands, Australia; University of Arizona, UNITED STATES

## Abstract

*Clostridium difficile* infection (CDI) is a leading cause of healthcare-associated morbidity and mortality worldwide. In Thailand, CDI exhibits low recurrence and mortality and its molecular epidemiology is unknown. CDI surveillance was conducted in a tertiary facility (Siriraj Hospital, Bangkok). A total of 53 toxigenic *C*. *difficile* strains from Thai patients were analyzed by multi-locus sequence typing (MLST), PCR ribotyping, and pulse-field gel electrophoresis (PFGE). The mean age of the cohort was 64 years and 62.3% were female; 37.7% of patients were exposed to > two antibiotics prior to a diagnosis of CDI, with beta-lactams the most commonly used drug (56.3%). Metronidazole was used most commonly (77.5%; success rate 83.9%), and non-responders were treated with vancomycin (success rate 100%). None of the isolates carried binary toxin genes. Most isolates (98.2–100%) were susceptible to metronidazole, vancomycin, tigecycline and daptomycin. There were 11 sequence types (STs), 13 ribotypes (RTs) and four PFGE types. Six previously identified STs (ST12, ST13, ST14, ST33, ST41 and ST45) and five novel STs unique to Thailand (ST66, ST67, ST68, ST69 and ST70) were identified. PCR RTs UK 017 (ST45) (45.3%) and UK 014/020 (ST33) (24.5%) were the most common. High concordance was observed between the MLST and ribotyping results (p<0.001). *C*. *difficile* isolates from Thai patients were highly susceptible to standard antimicrobial agents. In conclusion, the five STs indicate the high genetic diversity and unique polymorphisms in Thailand. Moreover, the emergence of antimicrobial resistance to vancomycin warranted continuous surveillance to prevent further spread of the toxigenic *C*. *difficile* isolates.

## Introduction

*Clostridium difficile*, an obligate anaerobic Gram-positive bacillus, is an important etiological agent of antibiotic-associated diarrhea (*C*. *difficile*-associated diarrhea; CDAD or *Clostridium difficile* infection; CDI). The disease is largely considered a healthcare-associated infection, with symptoms ranging from mild diarrhea to severe pseudomembranous colitis, which can occur during antibiotic treatment or shortly thereafter [1]. Two large clostridial toxins (LCTs), one an enterotoxin (toxin A; TcdA), the other a cytotoxin (toxin B; TcdB), are produced by toxigenic strains of *C*. *difficile*. The toxins interfere with the activity of proteins important for regulating actin polymerization, thereby leading to the disruption of the cell’s cytoskeleton [1]. Toxigenic strains capable of causing CDI produce both toxins; however, toxin A-negative strains are increasingly being reported [2–4].

An additional toxin discovered in 1997, the actin perturbing binary toxin (CDT), has been reported to be present in 4–12% of toxigenic *C*. *difficile* [5]. This toxin consists of two independent components comprising a catalytic (CDTa) and binding (CDTb) domain. Although the contribution of CDT to the virulence of *C*. *difficile* remains controversial, its biological function and its genetic and immunological properties are reported to closely resemble those of *Clostridium perfringens* iota toxin [6]. Interestingly, studies have reported synergy between CDT and LCTs [7,8], and that the presence of CDT might be an indicator for recurrent CDI [9].

As the prevalence of CDI increases globally as a consequence of high levels of antibiotic use, several techniques for investigating its epidemiology have been developed. Pulse-field gel electrophoresis (PFGE), PCR ribotyping and restriction endonuclease analysis have been used widely as typing methods [10]. Because gel banding patterns can be difficult to compare across laboratories, two multi-locus sequence typing (MLST) methods were recently established [11,12]. Use of the established MLST scheme for *C*. *difficile* and the publicly available online database of seven *C*. *difficile* housekeeping genes comprising *aroE* (shikimate dehydrogenase), *dutA* (dUTP pyrophosphatase), *gmk* (guanylate kinase), *groEL* (60 kDa chaperonin), *recA* (recombinase), *sodA* (superoxide dismutase) and *tpi* triosephosphate isomerase) enabled an inter-laboratory comparison of up to 65 of the sequence types (STs) present in the Pasteur Institute database [11]. The other MLST method, based on a different set of seven housekeeping genes, namely, *adk* (adenylate kinase), *atpA* (ATP synthase subunit alpha), *dxr* (1-deoxy-D-xylulose 5-phosphate reductoisomerase), *sodA* (superoxide dismutase), *recA* (recombinase A), *glyA* (serine hydroxymethyltransferase) and *tpi* (triose phosphate isomerase) had similar discriminatory power [12].

CDI is frequently reported to be associated with previous exposure of the bacterium to clindamycin, cephalosporins and fluoroquinolones. Treatment for CDI can include antibiotic discontinuation; however, this alone is not always effective and antibiotic treatment is necessary in moderate to severe cases [1]. Because the antibiotics recommended in standard treatment regimens are metronidazole and vancomycin [13], the susceptibilities of *C*. *difficile* strains to these agents have been investigated [14]. In most cases, a high degree of resistance was observed against antibiotics reported to be associated with CDI, and most strains were susceptible to the antibiotics recommended in the standard treatment regimen for this disease [14].

In Thailand, several studies have investigated the clinical and microbiological aspects of CDI [15–17]. In 1990, a study showed the prevalence of fecal toxin B was 52.5% among Thai diarrheal patients [15]. More recently, studies reported beta-lactam antibiotics to be the most commonly used drugs prior to the onset of CDAD [16]. Additionally, CDI was reported to have low recurrence and mortality rates [17]. However, at present, data regarding the molecular epidemiology and antibiotic susceptibility profile of Thai *C*. *difficile* isolates is limited. Thus, this study aimed to perform the first comprehensive epidemiological study of toxigenic *C*. *difficile* isolated from Thai patients.

## Materials and Methods

### Isolates and growth media

The 53 toxin-positive *C*. *difficile* strains used in this study were isolated from the stools of patients who were diagnosed with suspected CDI and had been admitted to Siriraj Hospital, Bangkok, Thailand, between 2006 and 2008. This study was approved by the Siriraj Institutional Review Board [approval no. Si414/2007]. The requirement for informed consents was waived as the study was a retrospective chart review. Patient records/information was anonymized and de-identified prior to analysis. Toxins in the isolates were detected with a qualitative immunochromatographic assay (Xpect *C*. *difficile* toxin A/B test; Thermo scientific, Lenexa, KS, USA), according to the manufacturer’s instructions. All toxin-positive samples identified in this manner were confirmed by toxin gene detection (as described below). Only toxin gene-positive strains were included in subsequent studies. Duplicate specimens from the same patients were excluded. Species identification was done according to internationally agreed standard testing procedures, which includes 16S ribosomal RNA sequence analysis [18]. Strains were maintained on blood agar plates at 37°C in anaerobic conditions and in 30% glycerol at –80°C for long term storage.

### Antibiotic susceptibility tests

Susceptibility of the toxigenic *C*. *difficile* strains to vancomycin, metronidazole, ciprofloxacin, moxifloxacin, linezolid, tigecycline and daptomycin was determined using an E-test as previously described [19]. The quality control strains for Gram-positive tests were *Staphylococcal aureus* ATCC 29213. Briefly, a suspension equivalent to a 0.5 McFarland standard for each isolate was prepared in nutrient broth (Oxoid Ltd, Basingstoke, UK) and swabbed onto supplemented (hemin and vitamin K1) Brucella agar containing 5% sheep blood (BBL Microbiology Systems, Cockeysville, MD). An E-test strip (AB Biodisk, Solna, Sweden) was applied to each plate and the plates were incubated at 37°C in anaerobic conditions for 48 h. The results were interpreted using the epidemiological cutoff values recommended by the European Committee on Antimicrobial Susceptibility Testing (EUCAST, http://www.eucast.org/) [20].

### DNA extraction

Genomic DNA was prepared from *C*. *difficile* grown on blood agar overnight at 37°C in anaerobic conditions. A full loop (approximately 10 μl) of cells was harvested and used for subsequent DNA extraction. High molecular weight DNA was extracted using a High Pure PCR Template Preparation Kit (Roche Applied Science, Indianapolis, IN) according to the manufacturer’s instructions.

### Detection of *C*. *difficile* toxin genes

*tcdA*, *tcdB*, *cdtA* and *cdtB* genes were detected in the isolates using a 5-plex PCR containing 12 primers specific to the four toxin genes and a control gene, 16S rDNA (S1 Table). The multiplex PCR assay was performed according to a previously published method [21]. Subsequently, the isolates were screened with an in-house PCR for the presence of toxin A (*tcdA*) [22] and for changes in the *tcdA* repetitive region [22].

### PCR ribotyping

Ribotyping of the isolates based on the 16S-23S intergenic spacer region was done as previously described [23]. PCR amplifications were performed using the following primers: 5’-CTGGGGTGAAGTCGTAACAAGG-3’ and 5’-GCGCCCTTTGTAGCTTGACC-3’. The PCR conditions comprised 35 cycles of denaturation at 94°C for 1 min, annealing at 55°C for 1 min and extension at 72°C for 2 min. Amplification products were concentrated and electrophoresis was performed on 3% agarose gels. The typing patterns generated were compared with those of a previously published library [10].

### PFGE

PFGE was performed according to a previously published method [24]. Briefly, the isolates were grown in pre-reduced Schaedler's anaerobic broth at 37°C overnight in an anaerobic atmosphere. The bacterial cells were pelleted and lysed in 100 μl of lysis buffer (10 mM Tris, 0.5 mM EDTA, 0.8% N-lauryl sarcosine, 5 mg/ml lysozyme). Plugs containing the bacterial cells were made using 2% PFGE-grade agarose with a low melting point. The bacterial plugs were then incubated in the lysis buffer for 1 h at 37°C followed by incubation in 0.5 mM EDTA, 1% N-lauryl sarcosine, 10 mg/ml proteinase K, overnight at 50°C. The plugs were washed and digested with *Sma*I restriction enzyme (20 U) for 5 h at 30°C. The digested products were run on a 1% PFGE-grade agarose gel using a CHEF Mapper (Bio-Rad, UK) with previously described settings [24]. The gels were documented and analyzed with a Fingerprinting II program (Bio-Rad). Dendrograms were constructed by the unweighted pair-group method with arithmetic mean clustering using the Dice correlation coefficient [25].

### MLST

MLST was performed and analyzed according to the methodologies of previous publications [11]. Briefly, seven unlinked genetic loci, *aroE* (shikimate dehydrogenase), *dutA* (dUTP pyrophosphatase), *gmk* (guanylate kinase), *groEL* (60 kDa chaperonin), *recA* (recombinase), *sodA* (superoxide dismutase) and *tpi* (triosephosphate isomerase) were amplified and sequenced. The sequences generated were manually edited using BioEdit 7.0.9.0, [25] and allele types (ATs) and STs for each locus were assigned according to the *C*. *difficile* MLST database at the Pasteur Institute (http://www.pasteur.fr/recherche/genopole/PF8/mlst/Cdifficile2.html) as previously described [12].

### Statistical analysis and correlation analysis

The PASW Statistics 18 program (IBM, Armonk, NY) was used to calculate frequencies of demographic data, antibiotic susceptibility, ATs and STs. Correlation analysis was done using a Lambda method for nominal variables by the PASW Statistics 18 program. Correlation and significance was defined when Lambda value was close to 1 and p-value was less than or equal to 0.05, respectively. Only MLST types with more than or equal to 3 members were subjected to the association analysis.

## Results

### *C*. *difficile* strains and patient demography

To obtain a complete overview of the epidemiology of pathogenic *C*. *difficile*, all strains isolated from 2006–2008 were tested for toxin production. Using immunochromatographic assays, a total of 53 *C*. *difficile* strains tested positive for toxin. The results of the multiplex PCR assay showed that these strains were also positive for *tcdA* and *tcdB*; therefore, they were included in this study. The cases from which *C*. *difficile* was isolated were aged 1–97 years, with a mean age of 64 years. The majority of patients were female (62.3%).

Analysis of the patient demography showed that 57.6% of the patients were being treated with antibiotics when CDI was diagnosed. We found that CDI can occur as late as 16 days post-antibiotic discontinuation (mean value of 2.52 days). As studies have shown that CDI can occur long after antibiotic exposure [1], here, we looked for a history of antibiotic use in the 2 months prior to diagnosis. During this period, patients with CDI were treated with zero to seven antibiotics, with 66.0% and 37.7% of them having used more than one and two antibiotics, respectively (S2 Table); 18.9% of the patients had no history of antibiotic use. Of the 126 antibiotic administration events, beta-lactams were the most frequently used drug (56.3%, Table 1).

**Table 1:** Antibiotic exposure in the 2 month period preceding a diagnosis of CDI in patients.

Antibiotic groups	Percent
**Beta-lactams** amoxicillin-clavulanic acid (0.8%), cefdinir (0.8%), cefditoren (0.8%), cefipime (4%), cefotaxime (1.6%), ceftazidime (5.6%), ceftriaxone (9.5%), cefuroxime (0.8%), cloxacillin (0.8%), ertapenem (1.6%), imipenem (10.3%), meropenem (5.6%), penicillin (0.8%), cefoperazone-sulbactam (2.4%), piperacillin-tazobactam (8.7%), ampicillin-sulbactam (2.4%)	56.3
**Glycopeptides** vancomycin (10.3%)	10.3
**Fluoroquinolones** ciprofloxacin (4.8%), levofloxacin (0.8%), moxifloxacin (1.6%), norfloxacin (0.8%)	7.9
**Aminoglycosides** amikacin (4%), netilmicin (0.8%)	4.8
**Metronidazole** metronidazole (4.8%)	4.8
**Lincosamides** clindamycin (4.8%)	4.8
**Others** amphotericin B (3.2%), colistin (2.4%), fluconazole (0.8%), fosfomycin (0.8%), ganciclovir (0.8%), itraconazole (0.8%), ivermectin (0.8%), roxithromycin (1.6%)	11.1

Metronidazole was the most common antimicrobial used for treatment (77.5%; 31 of 40 patients with available data) with a cure rate of 83.9% (26 of 31 patients with available data). Vancomycin was administered for 14 days in three cases where the patients failed to respond to metronidazole treatment; this resulted in a 100% cure rate (S2 Table). The other two patients died from complications; hence the efficacy of metronidazole could not be determined.

### Antibiotic susceptibility of *C*. *difficile*

The minimum inhibitory concentration (MIC) of each antibiotic (MIC_50_ and MIC_90_) is shown in Table 2. All *C*. *difficile* isolates were inhibited by a concentration of ≤ 0.5 μg/mL of metronidazole. All but two isolates were inhibited by a concentration of ≤ 0.75 μg/mL of vancomycin. Very high MICs (>32 μg/mL) were observed in all isolates tested with ciprofloxacin and in 43% of the isolates (23/53) tested with moxifloxacin. Most isolates exhibited low MICs (< 0.5 μg/mL) against linezolid, tigecycline and daptomycin (Table 2). Only moxifloxacin showed a considerable number of resistant isolates (45.2%) thus was subjected to correlation analysis. No correlation between the resistance with MLST, ribotype or PFGE type was observed (p-value = 0.818, 0.808 and 0.170, respectively).

**Table 2:** Susceptibility to various antibiotics of *C*. *difficile* isolates in this study (μg/mL).

Antibiotic	MIC (number of isolates)	% Susceptible*
Metronidazole	<0.016 (9), 0.016 (1), 0.023 (2), 0.032 (5), 0.047 (5), **0.064** (12), 0.094 (7), ***0*.*125*** (9), 0.19 (2), 0.5 (1)	100%
Vancomycin	0.016 (2), 0.023 (1), 0.038 (1), 0.047 (2), 0.064 (1), 0.094 (1), 0.125 (2), 0.25 (7), 0.38 (9), **0.5** (18), ***0*.*75*** (7), 1.5 (1), 3 (1)	98.2%
Ciprofloxacin	***>32*** ** (53)	NA
Moxifloxacin	0.032 (1), 0.25 (1), 0.38 (5), 0.5 (4), 0.75 (10), 1 (5), **1.5** (3), 24 (1), ***>32*** (23)	54.8%
Linezolid	0.023 (3), 0.032 (3), 0.047 (3), 0.064 (1), 0.094 (4), 0.125 (5), **0.19** (8), 0.25 (8), 0.38 (12), ***0*.*5*** (3), 0.75 (1), 1 (1), 2 (1)	NA
Tigecycline	**<0.016** (34), 0.016 (7), ***0*.*023*** (9), 0.032 (2), 0.064 (1)	100%
Daptomycin	0.023 (4), 0.032 (4), 0.047 (5), 0.064 (2), 0.094 (4), 0.125 (4), **0.19** (7), 0.25 (5), 0.38 (7), 0.5 (5), ***0*.*75*** (5), 1 (1)	100%

*Based on EUCAST epidemiological cutoff values (metronidazole ≤ 2 μg/mL, vancomycin ≤ 2 μg/mL, moxifloxacin ≤ 4 μg/mL, tigecycline ≤ 0.25 μg/mL, daptomycin ≤ 4 μg/mL) updated 14 Jan 2014 (http://www.eucast.org) [20].

**MIC_50_ and MIC_90 of_ ciprofloxacin are identical. Underlined bold numbers are MIC_50_, italicized bold numbers are MIC_90_. NA = not applicable.

### Molecular detection of *C*. *difficile* toxin

*tcdA* and *tcdB* genes were both present in all isolates, although 23 of them had a partial deletion in *tcdA* (A-B+). These included all strains from ribotypes (RTs) UK 017 (Th2, Th3, Th4, Th6, Th7, Th8, Th10, Th11, Th12, Th14, Th19, Th24, Th27, Th34, Th38, Th39, Th41, Th43, Th44, Th47, Th49 and Th53) and QX370 (Th45) (S2 Table). No isolates contained binary toxin genes (*cdtA* and *cdtB*).

### PFGE

**Fig 1:**
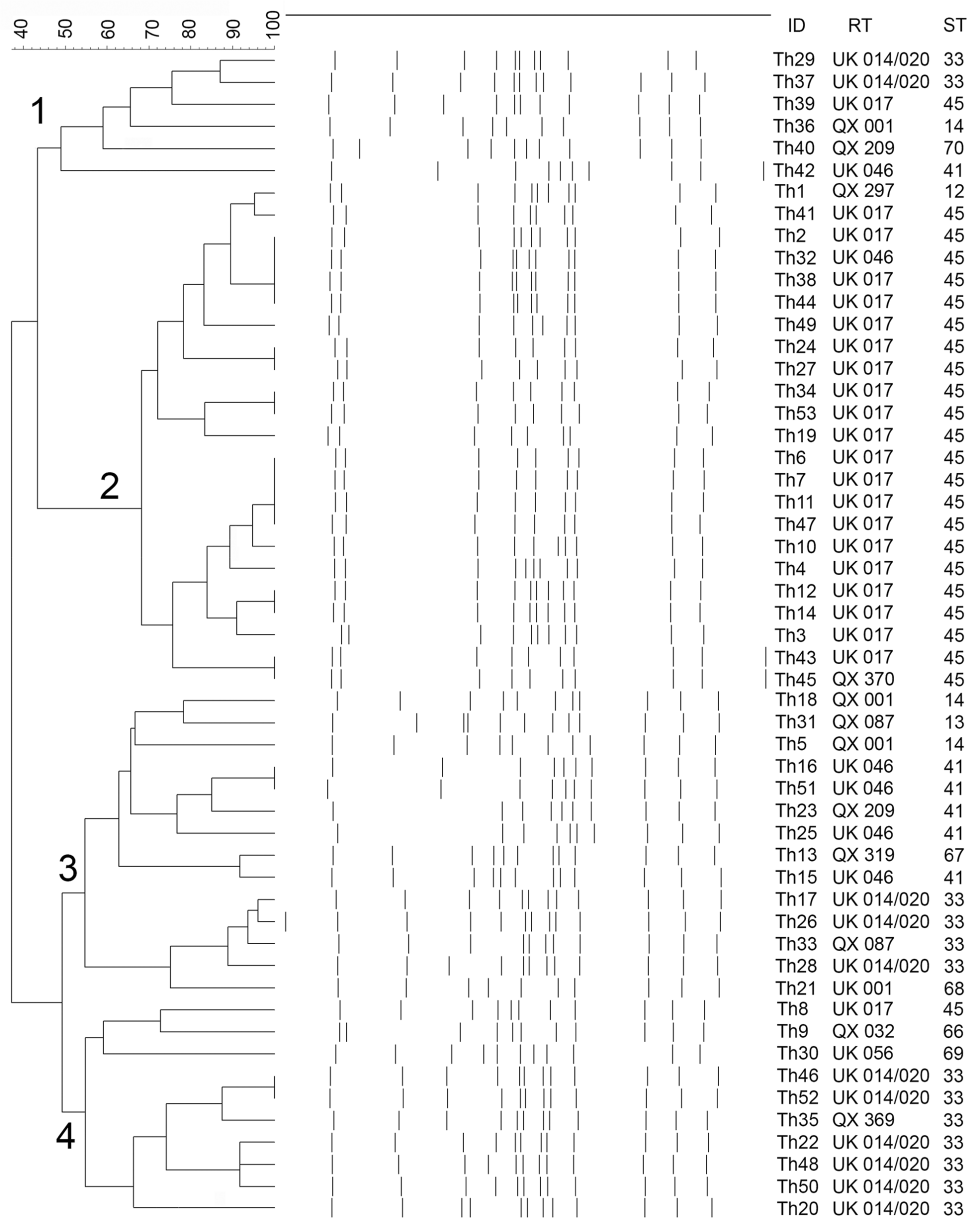
Dendrogram showing PFGE types and their correlations with RTs and STs. Numbers on the branches represent PFGE types. ID = strain identity; RT = ribotype; ST = sequence type. The scale represents the percentage level of similarity.

Four PFGE types were recognized with type 2 being the most common (43.4%, Fig 1).

### PCR ribotyping

Thirteen RTs were recognized. UK 017 (41.5%) and UK 014/020 (20.8%) were the two most common PCR RTs (Fig 1).

### MLST

Thirty ATs and 11 STs were recognized. Among the Thai *C*. *difficile* isolates, ST45 and ST33 were the most common (45.3%) and second most common (24.5%), respectively. Interestingly, all but one A-B+ strain (22 of 23) belonged to ST45. Additionally, one novel AT of the *groEL* gene (AT18) and five novel STs (ST66, ST67, ST68, ST69 and ST70) were also found. AT18 and the five novel ST sequences have been submitted to the *C*. *difficile* MLST database at the Pasteur Institute. The sequences of all the ATs have also been submitted to GenBank under the following accession numbers: KJ130417-446 (S3 Table).

### PCR ribotyping, PFGE and MLST method correlations

Overall, high concordance was observed between the ribotyping and two MLST methods, while PFGE categorized strains differently. For example, most strains of ST14, ST33, ST41 and ST45 correlates with the ribotype QX001 (100%), UK014/020 (84.6%), UK046 (83.3%) and UK017 (91.7%) with Lamda value of 0.87 (p-value <0.001). (Fig 1). However, RT UK 014/020 (ST33) correlated with PFGE types 1, 3 and 4 (Fig 1).

## Discussion

The risk factors associated with development of CDI include exposure to antibiotics, advanced age, hospitalization and residency in a long-term care facility [1]. Similar risk factors are also relevant in Thailand where most patients with CDI were of advanced age (mean age 64 years old), had been exposed to antibiotics and were long-term in-patients (96.2%; mean in-patient duration 62.4 days, data not shown). Analysis of patient medical records showed that beta-lactams were the most commonly administered antibiotics prior to the acquisition of CDI. This finding is consistent with the scientific literature, whereby antibiotics, including beta-lactams, lincosamides, and fluoroquinolones were frequently associated with CDI [26].

Among the 53 cases, two were young children aged 1 and 6 years old. Studies have found that *C*. *difficile* strains isolated from children with CDI are often acquired in the community in the absence of previous antibiotic exposure [27]. Although detailed medical records were unavailable, the low MICs of moxifloxacin (0.5 and 0.38 μg/mL) for the strains isolated from the two children indicate that their infections were acquired outside healthcare settings, particularly as the MICs were well-below the EUCAST epidemiological cut-off value (≤ 4 μg/mL) [28]. More research involving Thai pediatric cohorts is required before further conclusions can be drawn.

As metronidazole and vancomycin are the drugs of choice for CDI treatment [29], their MICs were determined. Although oral vancomycin and fidaxomicin are the only FDA-approved treatments for CDI, metronidazole has also been found to be effective at treating mild to moderate CDIs [13]. Symptom relief was observed in up to 20% of the cases within the first 48 h of following these standard treatments [30]. In Thailand, comparable results were observed for oral metronidazole (72.41%), while vancomycin was shown to be an effective treatment for CDI. Fluoroquinolone treatment is known to be a risk factor for the development of CDI [26]. As such, *C*. *difficile* isolates were tested for ciprofloxacin and moxifloxacin resistance. Finally, the MICs of various other antibiotics including linezolid, tigecycline and daptomycin against *C*. *difficile* were also investigated to determine their suitability as alternative treatments for CDI [14,30]. As anticipated, most *C*. *difficile* strains exhibited high MICs when tested against fluoroquinolones, particularly ciprofloxacin, while low MICs were observed with the new antibiotics, suggesting their potential as new therapeutic options for CDI.

Because CDT was previously found in 4–12% of toxigenic *C*. *difficile* samples [5], the isolates were screened for the presence of *cdtA* and *cdtB*. Despite CDT-producers increasingly being reported as the causative agents of CDI, our results suggest that this has not occurred in Thailand. Infection with CDT-positive *C*. *difficile* is associated with higher mortality and recurrence rates [9]. This is in agreement with our findings that the CDT-negative *C*. *difficile* isolated from Thai patients exhibited low virulence and no resistance against standard drug treatments. Moreover, as reported previously [18], the A-B+ variant isolates mostly belonged to the same genotype (UK 017 and ST45). This emphasizes the high correlation observed between the standard genotyping methods and the toxin genotype.

As anticipated from the treatment outcomes, the isolates had high susceptibility levels against metronidazole and vancomycin, which are the first-line drugs for CDI treatment. Similarly, no resistance was observed against the alternative drugs, tigecycline and daptomycin. For linezolid, although an epidemiological cut-off value has not been established, all isolates showed promising MICs of ≤ 2 μg/mL, which is a clinical breakpoint for streptococci [20], and lower than those isolated from the United States where strains with MIC as high as 8 μg/mL were isolated from both animals [31] and humans [32]. Additionally, the high MIC values of the isolates against fluoroquinolones was as expected, given the existing scientific literature. Overall, the antibiotic susceptibility of Thai *C*. *difficile* isolates is in line with surveys from Sweden [14], Taiwan [19] and Canada [33], where high susceptibility to metronidazole and vancomycin, and low susceptibility to fluoroquinolones were reported. However, as the clinical breakpoints for these agents have not been established for *C*. *difficile*, interpretation of the MIC values may produce misleading results, and their clinical applications are limited.

Finally, because MLST and ribotyping methods typically exhibit high discriminatory power and are reliable and reproducible, they were used in this study. High overall agreement was observed between the two techniques, which further emphasized their reliability and reproducibility. Six of the STs (ST12, ST13, ST14, ST33, ST41 and ST45) among the isolates have also been reported in Europe, North America and Asia [11,34]. The two most common STs in our study (ST45 and ST33) were also among the most common STs in the MLST database containing 539 isolates [34]. Similarly, the rare STs in our study (ST12, ST13 and ST14) were also rare in this database. Interestingly, the hypervirulent PCR RT UK 027 (ST3) [35] was not observed. The absence of the latter and other CDT-producers might have contributed to the low recurrence and mortality rates observed in Thailand thus far [17]. However, to be able to draw this conclusion, further sampling of C. difficile from other area in Thailand is warranted.

## Supporting Information

S1 Table Primers used in this study. (DOCX)

S2 Table Patient demographic data. (DOCX)

S3 Table GenBank accession numbers for the sequences of the MLST alleles. (DOCX)
