# Correction: Molecular Epidemiology of *Clostridium difficile* Infection in a Large Teaching Hospital in Thailand

**DOI:** 10.1371/journal.pone.0134771

**Published:** 2015-07-29

**Authors:** Popchai Ngamskulrungroj, Sittinee Sanmee, Papanin Putsathit, Pipat Piewngam, Briony Elliott, Thomas V. Riley, Pattarachai Kiratisin

The third author’s name is spelled incorrectly. The correct name is: Papanin Putsathit. The correct citation is: Ngamskulrungroj P, Sanmee S, Putsathit P, Piewngam P, Elliott B, Riley TV, et al. (2015) Molecular Epidemiology of *Clostridium difficile* Infection in a Large Teaching Hospital in Thailand. PLoS ONE 10(5): e0127026. doi:10.1371/journal.pone.0127026
