# Supplementary material for: Molecular Epidemiology of Clostridium difficile Infection in a Large Teaching Hospital in Thailand
Source: PLoS One. 2015 May 22;10(5):e0127026. doi: 10.1371/journal.pone.0127026 (PMC4441498; doi:10.1371/journal.pone.0127026)
Supplement: S1 Table — (DOCX) [file pone.0127026.s001.docx]

**Table S1.** Primers used in this study

| Genes/fragments | Primers |
| --- | --- |
| *tcdA* | tcdA-F3345: GCATGATAAGGCAACTTCAGTGGTA  tcdA-R3969: AGTTCCTCCTGCTCCATCAAATG |
| *tcdB* | tcdB-F5670: CCAAARTGGAGTGTTACAAACAGGTG  tcdB-R6079A: GCATTTCTCCATTCTCAGCAAAGTA  tcdB-R6079B: GCATTTCTCCGTTTTCAGCAAAGTA |
| *cdtA* | cdtA-F739A: GGGAAGCACTATATTAAAGCAGAAGC  cdtA-F739B: GGGAAACATTATATTAAAGCAGAAGC  cdtA-R958: CTGGGTTAGGATTATTTACTGGACCA |
| *cdtB* | ctdB-F617: TTGACCCAAAGTTGATGTCTGATTG  cdtB-R878: CGGATCTCTTGCTTCAGTCTTTATAG |
| *aroE* | aroE-F : CTAGTAGGTGAAAAACTCTCTCA  aroE-R : ACTGGTGTAGCATTTAATATTATATC |
| *dutA* | dutA-F : CCTAATTTTGCTCACAAAGGT  dutA-R : AAATCCAGTTGAGCCAAACC |
| *gmk* | gmk-F : AGGTGCAGGAAAAGGTACTAT  gmk-R : TTCATATGCACAGCTAAATCTC |
| *­groEL* | groEL-F : ATACTGAATTAGATGCTGTTGAAG  groEL-R : TCTGAAGTAGTTTGCTCTACTTG |
| *recA* | recA2F: CAGTAATGAAATTGGGAGAAGC  recA2R: ATTCAGCTTGCTTAAATGGTG |
| *sodA* | sodA5F: CCAGTTGTCAATGTATTCATTTC  sodA6R: ATAACTTCATTTGCTTTTACACC |
| *tpi* | tpi2F: ATGAGAAAACCTATAATTGCAG  tpi2R: TTGAAGGTTTAACACT |
| *tcdA* | NK3: GGAAGAAAAGAACTTCTGGCTCACTCAGGT  NK2: CCCAATAGAAGATTCAATATTAAGCTT |
| *tcdA** | NK9: CCACCAGCTGCAGCCATA  NK11: TGATGCTAATAATGAATCTAAAATGGTAAC |
| *tcdB* | NK104: GTGTAGCAATGAAAGTCCAAGTTTACGC  NK105: CACTTAGCTCTTTGATTGCTGCACCT |
| 16S rRNA | PS13: GGAGGCAGCAGTGGGGAATA  PS14: TGACGGGCGGTGTGTACAAG |

*for detection of *tcdA* partial deletion according to changes in repeating region of *tcdA*
