# Supplementary material for: Molecular Epidemiology of Clostridium difficile Infection in a Large Teaching Hospital in Thailand
Source: PLoS One. 2015 May 22;10(5):e0127026. doi: 10.1371/journal.pone.0127026 (PMC4441498; doi:10.1371/journal.pone.0127026)
Supplement: S2 Table — (DOCX) [file pone.0127026.s002.docx]

**Table S2.** Patient demographic data

| **Strain**  **ID** | **Diagnosis** | **Number**  **of**  **antibiotics**  **used*** | **Treatment** | **Treatment**  **duration** | **Toxin A and B PCR** | **AT** | | | | | | | **ST** |
| --- | --- | --- | --- | --- | --- | --- | --- | --- | --- | --- | --- | --- | --- |
|  |  |  |  |  |  | ***aroE*** | ***dutA*** | ***gmk*** | ***groEL*** | ***recA*** | ***sodA*** | ***Tpi*** |  |
| Th1 | n | 1 | Vancomycin** | 14 | A+B+ | 7 | 6 | 1 | 6 | 2 | 6 | 1 | 12 |
| Th2 | n | 0 | n | n | A-B+ | 2 | 2 | 2 | 2 | 2 | 2 | 2 | 45 |
| Th3 | n | 0 | n | n | A-B+ | 2 | 2 | 2 | 2 | 2 | 2 | 2 | 45 |
| Th4 | Cancer | 2 | metronidazole | 7 | A-B+ | 2 | 2 | 2 | 2 | 2 | 2 | 2 | 45 |
| Th5 | Bedridden  illness | 1 | metronidazole | 7 | A+B+ | 5 | 1 | 3 | 6 | 5 | 6 | 1 | 14 |
| Th6 | n | 0 | n | n | A-B+ | 2 | 2 | 2 | 2 | 2 | 2 | 2 | 45 |
| Th7 | Infection | 4 | metronidazole | 7 | A-B+ | 2 | 2 | 2 | 2 | 2 | 2 | 2 | 45 |
| Th8 | n | 2 | metronidazole | 7 | A-B+ | 2 | 2 | 2 | 2 | 2 | 2 | 2 | 45 |
| Th9 | CA bladder | 2 | no | n | A+B+ | 5 | 6 | 1 | 6 | 1 | 3 | 1 | 66 |
| Th10 | Infection | 1 | metronidazole |  | A-B+ | 2 | 2 | 2 | 2 | 2 | 2 | 2 | 45 |
| Th11 | Infection | 4 | metronidazole | 5 | A-B+ | 2 | 2 | 2 | 2 | 2 | 2 | 2 | 45 |
| Th12 | n | 0 | n | n | A-B+ | 2 | 2 | 2 | 2 | 2 | 2 | 2 | 45 |
| Th13 | Cancer | 2 | no | n | A+B+ | 5 | 1 | 3 | 3 | 1 | 6 | 3 | 67 |
| Th14 | Cancer | 3 | metronidazole | 5 | A-B+ | 2 | 2 | 2 | 2 | 2 | 2 | 2 | 45 |
| Th15 | Infection | 4 | no | n | A+B+ | 5 | 6 | 3 | 3 | 1 | 1 | 7 | 41 |
| Th16 | Infection | 1 | Vancomycin** | 14 | A+B+ | 5 | 6 | 3 | 3 | 1 | 1 | 7 | 41 |
| Th17 | Cancer | 4 | Vancomycin**  /cholestyramine | n | A+B+ | 1 | 1 | 3 | 3 | 3 | 1 | 3 | 33 |
| Th18 | Bedridden  illness | 3 | metronidazole | 7 | A+B+ | 5 | 1 | 3 | 6 | 5 | 6 | 1 | 14 |
| Th19 | Infection | 2 | no | n | A-B+ | 2 | 2 | 2 | 2 | 2 | 2 | 2 | 45 |
| Th20 | Cancer | 1 | metronidazole | 7 | A+B+ | 1 | 1 | 3 | 3 | 3 | 1 | 3 | 33 |
| Th21 | n | 2 | piperacillin  /tazobactam | n | A+B+ | 18 | 6 | 1 | 18 | 3 | 6 | 3 | 68 |
| Th22 | Bedridden  illness | 2 | metronidazole | 7 | A+B+ | 1 | 1 | 3 | 3 | 3 | 1 | 3 | 33 |
| Th23 | Infection | 4 | metronidazole | 9 | A+B+ | 5 | 6 | 3 | 3 | 1 | 1 | 7 | 41 |
| Th24 | n | 2 | metronidazole | 10 | A-B+ | 2 | 2 | 2 | 2 | 2 | 2 | 2 | 45 |
| Th25 | Cancer | 1 | metronidazole | n | A+B+ | 5 | 6 | 3 | 3 | 1 | 1 | 7 | 41 |
| Th26 | Infection | 3 | metronidazole | 7 | A+B+ | 1 | 1 | 3 | 3 | 3 | 1 | 3 | 33 |
| Th27 | Bedridden  illness | 2 | metronidazole | 7 | A-B+ | 2 | 2 | 2 | 2 | 2 | 2 | 2 | 45 |
| Th28 | n | 0 | n | n | A+B+ | 1 | 1 | 3 | 3 | 3 | 1 | 3 | 33 |
| Th29 | n | 0 | n | n | A+B+ | 1 | 1 | 3 | 3 | 3 | 1 | 3 | 33 |
| Th30 | Infection | 2 | metronidazole | 10 | A+B+ | 7 | 1 | 6 | 3 | 1 | 1 | 3 | 69 |
| Th31 | Infection | 2 | no | n | A+B+ | 6 | 1 | 3 | 6 | 5 | 6 | 3 | 13 |
| Th32 | Cancer | 3 | metronidazole | 8 | A+B+ | 2 | 2 | 2 | 2 | 2 | 2 | 2 | 45 |
| Th33 | Infection | 4 | metronidazole | n | A+B+ | 1 | 1 | 3 | 3 | 3 | 1 | 3 | 33 |
| Th34 | Infection | 3 | Vancomycin** | 14 | A-B+ | 2 | 2 | 2 | 2 | 2 | 2 | 2 | 45 |
| Th35 | Bedridden  illness | 2 | Vancomycin** | n | A+B+ | 1 | 1 | 3 | 3 | 3 | 1 | 3 | 33 |
| Th36 | Bedridden  illness | 5 | n | 7 | A+B+ | 5 | 1 | 3 | 6 | 5 | 6 | 1 | 14 |
| Th37 | Miscellaneous | 2 | erythromycin | n | A+B+ | 1 | 1 | 3 | 3 | 3 | 1 | 3 | 33 |
| Th38 | Miscellaneous | 3 | metronidazole | 11 | A-B+ | 2 | 2 | 2 | 2 | 2 | 2 | 2 | 45 |
| Th39 | Miscellaneous | 4 | metronidazole | 7 | A-B+ | 2 | 2 | 2 | 2 | 2 | 2 | 2 | 45 |
| Th40 | Infection | 4 | metronidazole | 7 | A+B+ | 2 | 2 | 3 | 1 | 2 | 1 | 2 | 70 |
| Th41 | Infection | 7 | no | n | A-B+ | 2 | 2 | 2 | 2 | 2 | 2 | 2 | 45 |
| Th42 | Infection | 1 | no | n | A+B+ | 5 | 6 | 3 | 3 | 1 | 1 | 7 | 41 |
| Th43 | Cancer | 4 | metronidazole | 7 | A-B+ | 2 | 2 | 2 | 2 | 2 | 2 | 2 | 45 |
| Th44 | Infection | 2 | metronidazole | n | A-B+ | 2 | 2 | 2 | 2 | 2 | 2 | 2 | 45 |
| Th45 | Cancer | 4 | metronidazole | n | A-B+ | 2 | 2 | 2 | 2 | 2 | 2 | 2 | 45 |
| Th46 | Infection | 1 | metronidazole | 14 | A+B+ | 1 | 1 | 3 | 3 | 3 | 1 | 3 | 33 |
| Th47 | Miscellaneous | 3 | metronidazole | 3 | A-B+ | 2 | 2 | 2 | 2 | 2 | 2 | 2 | 45 |
| Th48 | n | 0 | n | n | A+B+ | 1 | 1 | 3 | 3 | 3 | 1 | 3 | 33 |
| Th49 | n | 0 | n | n | A-B+ | 2 | 2 | 2 | 2 | 2 | 2 | 2 | 45 |
| Th50 | Infection | 2 | n | n | A+B+ | 1 | 1 | 3 | 3 | 3 | 1 | 3 | 33 |
| Th51 | Infection | 4 | n | n | A+B+ | 5 | 6 | 3 | 3 | 1 | 1 | 7 | 41 |
| Th52 | n | 0 | n | n | A+B+ | 1 | 1 | 3 | 3 | 3 | 1 | 3 | 33 |
| Th53 | n | 0 | n | n | A-B+ | 2 | 2 | 2 | 2 | 2 | 2 | 2 | 45 |

* In the last 2 months; ** started after failures of metronidazole treatments; n = no data
