# Supplementary material for: Molecular Epidemiology of Clostridium difficile Infection in a Large Teaching Hospital in Thailand
Source: PLoS One. 2015 May 22;10(5):e0127026. doi: 10.1371/journal.pone.0127026 (PMC4441498; doi:10.1371/journal.pone.0127026)
Supplement: S3 Table — (DOCX) [file pone.0127026.s003.docx]

**Table S3.** GenBank accession numbers for the sequences of the MLST alleles

| **Species** | **Loci** | **Alleles** | **GenBank Accession number** |
| --- | --- | --- | --- |
| *C. difficile* | aroE | aroE allele 1 | KJ130417 |
|  |  | aroE allele 2 | KJ130418 |
|  |  | aroE allele 5 | KJ130419 |
|  |  | aroE allele 6 | KJ130420 |
|  |  | aroE allele 7 | KJ130421 |
|  |  | aroE allele 18 | KJ130422 |
|  | dutA | dutA allele 1 | KJ130423 |
|  |  | dutA allele 2 | KJ130424 |
|  |  | dutA allele 6 | KJ130425 |
|  | gmk | gmk allele 1 | KJ130426 |
|  |  | gmk allele 2 | KJ130427 |
|  |  | gmk allele 3 | KJ130428 |
|  |  | gmk allele 6 | KJ130429 |
|  | groEL | groEL allele 1 | KJ130430 |
|  |  | groEL allele 2 | KJ130431 |
|  |  | groEL allele 3 | KJ130432 |
|  |  | groEL allele 6 | KJ130433 |
|  |  | groEL allele 18 | KJ130434 |
|  | recA | recA allele 1 | KJ130435 |
|  |  | recA allele 2 | KJ130436 |
|  |  | recA allele 3 | KJ130437 |
|  |  | recA allele 5 | KJ130438 |
|  | sodA | sodA allele 1 | KJ130439 |
|  |  | sodA allele 2 | KJ130440 |
|  |  | sodA allele 3 | KJ130441 |
|  |  | sodA allele 6 | KJ130442 |
|  | tpi | tpi allele 1 | KJ130443 |
|  |  | tpi allele 2 | KJ130444 |
|  |  | tpi allele 3 | KJ130445 |
|  |  | tpi allele 7 | KJ130446 |
